# *Notes from the Field:* Lead Exposures Among Employees at a Bullet Manufacturing Company — Missouri, 2017

**DOI:** 10.15585/mmwr.mm6739a7

**Published:** 2018-10-05

**Authors:** David A. Jackson, Gregory A. Burr, Carol R. Braun, Marie A. de Perio

**Affiliations:** ^1^Epidemic Intelligence Service, CDC; ^2^Division of Surveillance, Hazard Evaluations, and Field Studies, National Institute for Occupational Safety and Health, CDC; ^3^Missouri Department of Health and Senior Services.

Lead is toxic to all human organ systems, resulting in adverse health effects that include impaired kidney function, elevated blood pressure, and neurologic health effects (*1*). Lead primarily enters the body through inhalation and ingestion, but direct absorption through the skin can occur (*2*). According to 2014 national lead surveillance data, >94% of the 3,616 U.S. adults with elevated blood lead levels (BLLs) whose exposure source was known were exposed at work (*3*).

Because of concerns about employees’ occupational lead exposures, a Missouri bullet manufacturing company that melts lead ingots and casts them into bullets asked CDC’s National Institute for Occupational Safety and Health (NIOSH) to conduct a health hazard evaluation. In October 2017, NIOSH visited the worksite to determine the routes and extent of lead exposure among employees and the prevalence of elevated BLLs and to assess controls in place to protect employees from lead exposure.

Full-shift personal air samples and blood specimens for lead were collected from 10 of 11 employees. All 11 employees were interviewed and provided lead hand wipe samples before lunch and at the end of their work shift after washing their hands. Work practices and conditions were also observed. An elevated BLL was defined as ≥5 *μ*g/dL, the CDC adult blood lead reference level (*3*,*4*). Lead air sample results were compared with occupational exposure limits.

Among 10 tested employees, the median BLL was 8.5 *μ*g/dL (range = 4–35 *μ*g/dL). Of these employees, nine had an elevated BLL, including packaging and shipping employees. The three employees with the highest BLLs worked in the casting and coating areas. All lead air concentrations were below the Occupational Safety and Health Administration (OSHA) permissible exposure limit of 50 *μ*g/m^3^ of air. Lead air concentrations measured in the casting and coating areas were the highest. All employees had lead on their hands after washing them. Interviews revealed inconsistent glove use and handwashing with lead removal soap and lack of clothes or shoes dedicated only to use at the worksite, as well as reports of dry sweeping the floors. Food and beverages were observed in work areas. Skin lesions were observed on the arms of casting area employees, who reported that these lesions were caused by molten lead.

Almost all employees at this worksite had elevated BLLs. Although personal airborne lead exposures were below the OSHA permissible exposure limit, lack of a workplace lead control program likely resulted in employee lead exposures through inhalation, ingestion, and dermal absorption. Education and training to improve work practices are needed to reduce employee lead exposures. Such improved work practices would include consistently wearing disposable nonlatex gloves in bullet production areas and handwashing with lead removal soap; using HEPA-filtered vacuums for surface cleaning; eliminating food and drink storage and consumption from bullet production areas; and wearing heat-resistant gloves, sleevelets, or both in the casting area to protect skin. The company was also advised to start a comprehensive lead program that incorporates elements of the OSHA lead standard, including training of workers and medical surveillance (*5*).
